# Massive Upper Gastrointestinal Bleeding From a Mediastinal Tuberculous Esophageal Fistula

**DOI:** 10.7759/cureus.100204

**Published:** 2025-12-27

**Authors:** Nada Faquir, Fatimazahra Belabbes, Nawal Bouknani, Amal Rami, Imane Ben Elbarhdadi

**Affiliations:** 1 Gastroenterology and Proctology, Cheikh Khalifa International University Hospital, Mohammed VI University of Health Sciences, Casablanca, MAR; 2 Radiology, Cheikh Khalifa International University Hospital, Mohammed VI University of Health Sciences, Casablanca, MAR; 3 Gastroenterology and Hepatology, Cheikh Khalifa International University Hospital, Mohammed VI University of Health Sciences, Casablanca, MAR

**Keywords:** endoscopic ultrasound (eus), esophageal ulcer, mediastinal tuberculosis, transarterial embolization, upper gastrointestinal bleeding

## Abstract

Upper gastrointestinal bleeding (UGIB) is a common medical emergency, most often caused by peptic ulcer disease, variceal rupture, or severe erosive mucosal injury. However, rare etiologies like vascular malformations or infectious processes, including tuberculosis, may also result in severe and life-threatening hemorrhage. Mediastinal tuberculosis with esophageal involvement is an exceptionally rare cause of UGIB.

We report a case of a 43-year-old man admitted for massive hematemesis and hemodynamic instability. Endoscopy revealed a large ulcer in the mid-esophagus with active bleeding. Endoscopic ultrasound and contrast-enhanced CT demonstrated a complex esophago-mediastinal lesion with fistulous communication and a pseudoaneurysm of the right bronchial artery. Despite multiple non-diagnostic endoscopic and surgical biopsies, endobronchial ultrasound-guided transbronchial needle aspiration (EBUS-TBNA) identified necrotizing granulomatous inflammation consistent with tuberculosis. Transarterial embolization was performed successfully to control bleeding, followed by initiation of standard anti-tuberculosis therapy. The patient’s clinical and radiological outcomes were favorable over follow-up.

Mediastinal tuberculosis with esophageal extension is a rare but important differential diagnosis in patients presenting with unexplained UGIB. This case highlights the diagnostic value of EBUS-TBNA in identifying deep mediastinal tuberculosis when conventional biopsies are inconclusive and the role of transarterial embolization as an effective hemostatic intervention. Early multidisciplinary management is essential to ensure both etiologic treatment and prevention of recurrence.

## Introduction

Upper gastrointestinal bleeding (UGIB) is a frequent and potentially life-threatening medical emergency, accounting for a significant proportion of hospital admissions in gastroenterology. It is classically manifested by hematemesis, melena, or both, and remains associated with considerable morbidity and mortality despite advances in diagnostic and therapeutic strategies. Early recognition and prompt management are therefore essential to improve clinical outcomes. The majority of UGIB cases are attributable to well-recognized etiologies, including peptic ulcer disease, esophageal varices related to portal hypertension, and inflammatory conditions like severe gastritis or esophagitis. These causes are widely described in the literature and are routinely encountered in clinical practice, allowing for standardized diagnostic and therapeutic approaches [1].

In contrast, UGIB may rarely result from unusual and unexpected conditions, including mediastinal vascular malformations, arterial aneurysms, and aorto-digestive fistulas, which are often associated with severe or massive hemorrhage and pose significant diagnostic challenges. Among these rare etiologies, mediastinal tuberculosis with secondary esophageal involvement represents an exceptionally uncommon but clinically important cause of UGIB. Tuberculosis is a multisystem disease with diverse clinical presentations; however, esophageal involvement is rare and typically occurs through contiguous spread from mediastinal lymphadenopathy. When present, it may lead to ulceration, fistulization, or bleeding, thereby mimicking more common causes of UGIB and delaying diagnosis [2].

We report a case of a 43-year-old man admitted for massive hematemesis in whom endoscopic and radiological evaluations revealed an esophageal ulcer complicated by a mediastinal fistulous tract. The etiologic diagnosis ultimately established mediastinal tuberculosis with esophageal extension, a rare but severe manifestation of extrapulmonary tuberculosis.

## Case presentation

**Figure 1:**
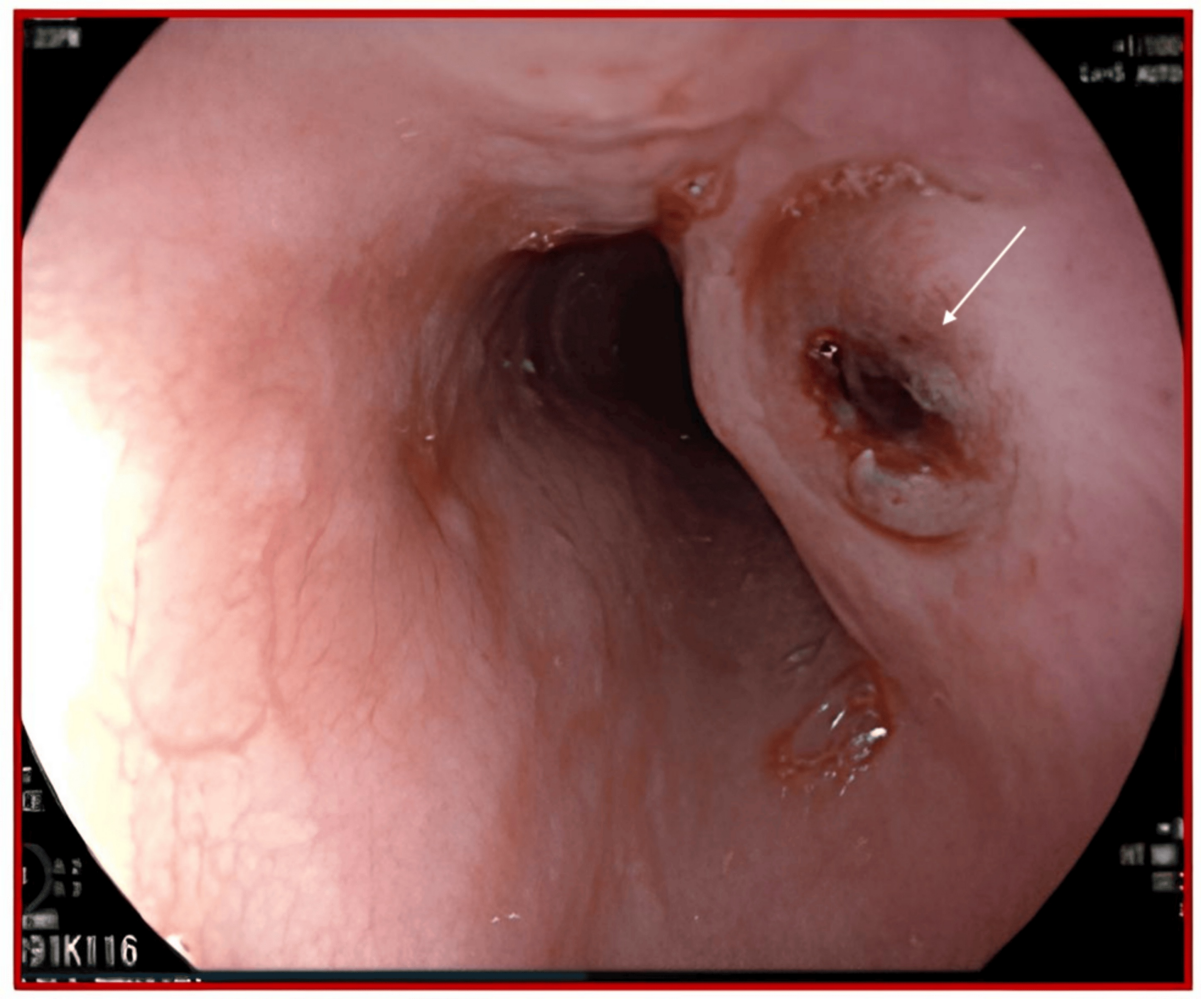
Endoscopy image of the esophageal ulcer. The esophageal mucosa appears generally inflamed and congested. On the right lateral wall, there is a rounded ulcerated lesion with irregular, raised edges and a whitish fibrino-necrotic base in some areas (white arrow).

A 43-year-old male patient with no previous medical history, immunocompetent without HIV, was admitted to the emergency room for profuse hematemesis, complicated by hemodynamic instability and blood loss, with a hemoglobin level of 6.2 g/dL. After stabilization and treatment in the emergency department, an emergency esophagogastroduodenal endoscopy was performed, revealing a large esophageal ulcer - approximately 30 cm from the dental arches, 3 cm deep, with raised edges - the bottom of which was occupied by a blood clot and presented bleeding (Figure 1).

**Figure 2:**
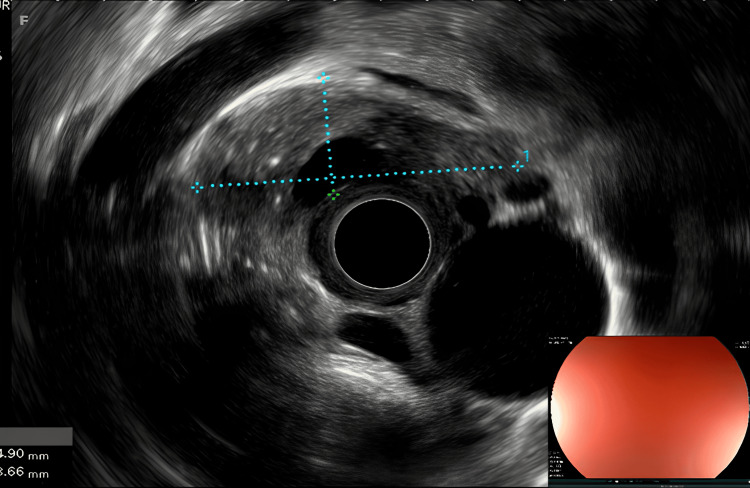
Endoscopic ultrasound of the ulcer showing hemicircular thickening of the esophageal wall.

An additional echoendoscopy showed a hemicircular thickening of the esophageal wall between 30 and 34 cm from the dental arches, heterogeneous, containing a cystic pocket measuring 36 x 13 mm, with interruption of the parietal layers and the presence of intradural air bubbles. This formation encompassed adjacent vascular branches and was accompanied by multiple subcentimeter lymphadenopathies - subcarinal and subaortic. The appearance was suggestive of an ulcerated formation complicated by a fistulous tract (Figure 2). 

A thoraco-abdominal CT scan confirmed the presence of a mediastinal process with fistula formation in the esophagus, with no signs of an aneurysmal sac. It revealed poorly defined tissue infiltration of the middle mediastinum, located between the D6 and D8 vertebrae, isodense, enhanced after contrast injection, measuring 17 mm at its thickest point and extending 32 mm in height, encompassing the esophagus and carina. This hypervascularized formation was supplied by two dilated bronchial arteries, with the presence of a false aneurysm of the right bronchial artery measuring 10 × 8 mm.

**Figure 3:**
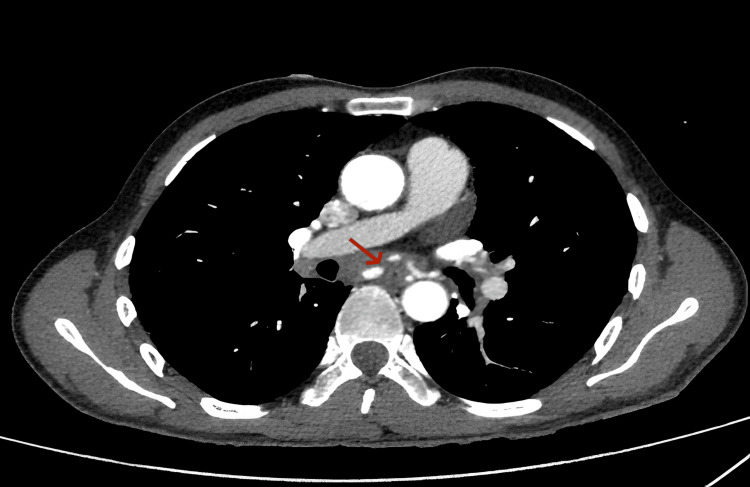
CT scan with injection showing bleeding (red arrow).

**Figure 4:**
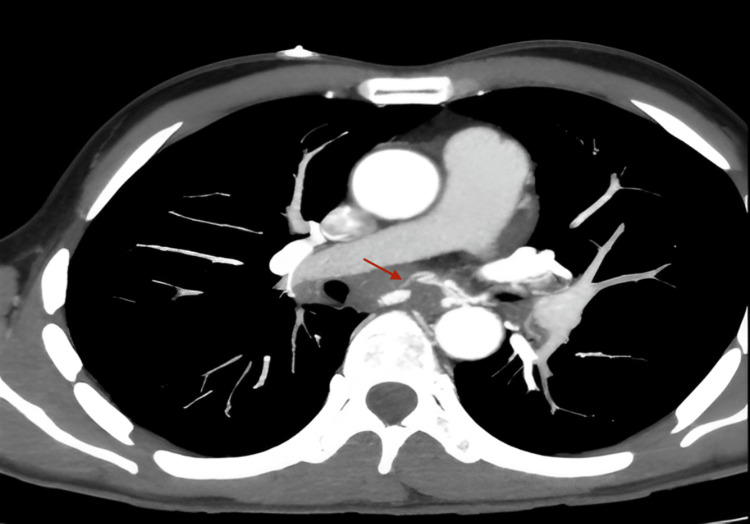
CT scan without injection in the arterial phase showing esophageal bleeding. This is an axial contrast-enhanced CT slice at the level of the pulmonary arteries. The red arrow points to a focal area of contrast leakage adjacent to a pulmonary artery. This hyperdense area outside the vessel indicates active hemorrhage.

**Figure 5:**
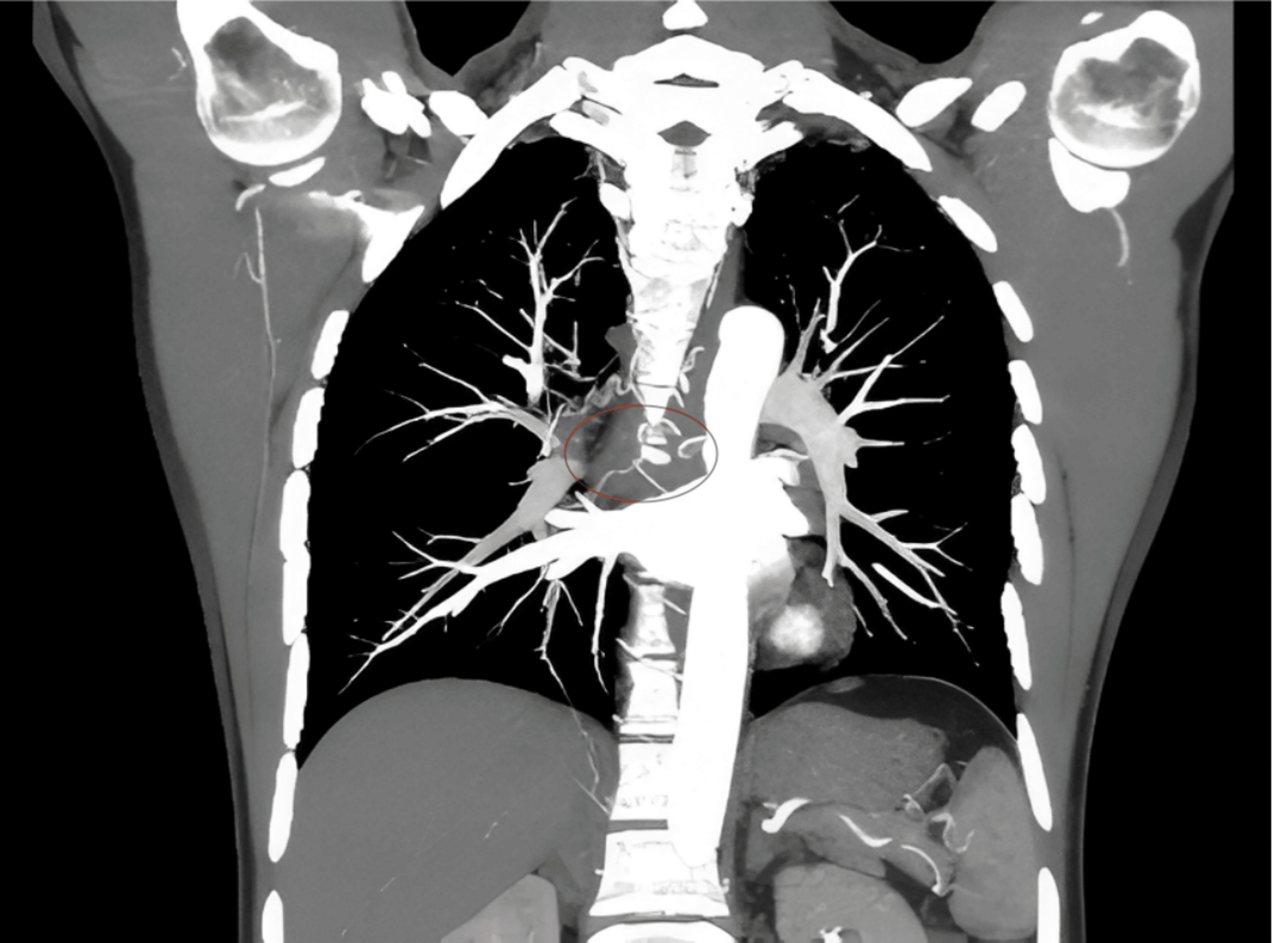
CT scan of the chest showing active contrast extravasation from the mid-thoracic esophagus (encircled), indicating ongoing active bleeding. The pulmonary arteries are opacified with contrast. There is an area of contrast extravasation near the pulmonary vessels, meaning that contrast is seen outside the normal vessel lumen, which is abnormal and consistent with active bleeding.

Anteriorly, it was in contact with the right atrium and the right branch of the pulmonary artery, with no separating border. Posteriorly, it was in contact with the descending thoracic aorta over 180°, also with no separating border (Figures 3-5). 

Repeated endoscopic biopsies were inconclusive, revealing fibrosis and necrotic material without specificity. A thoracoscopy was then performed, with mediastinal dissection and biopsies of several lateral tracheal and subcarinal lymph nodes, which were also non-specific. The patient was then referred for bronchial echoendoscopy, which revealed several lymph nodes, particularly subcarinal, as well as a bronchial tumor bud.

Pathological examination of the lymph node samples showed fibrin-hematic material rich in plasma cells, neutrophils, multinucleated giant cells, and epithelioid clusters. Samples taken from the tumor revealed a polypoid mucosa with a dense inflammatory chorion rich in polymorphic cells, including two epithelioid giant cell granulomas with foci of caseous necrosis punctuated by altered neutrophils. The overall picture was consistent with tuberculoid granulomatous inflammation compatible with tuberculosis.

**Figure 6:**
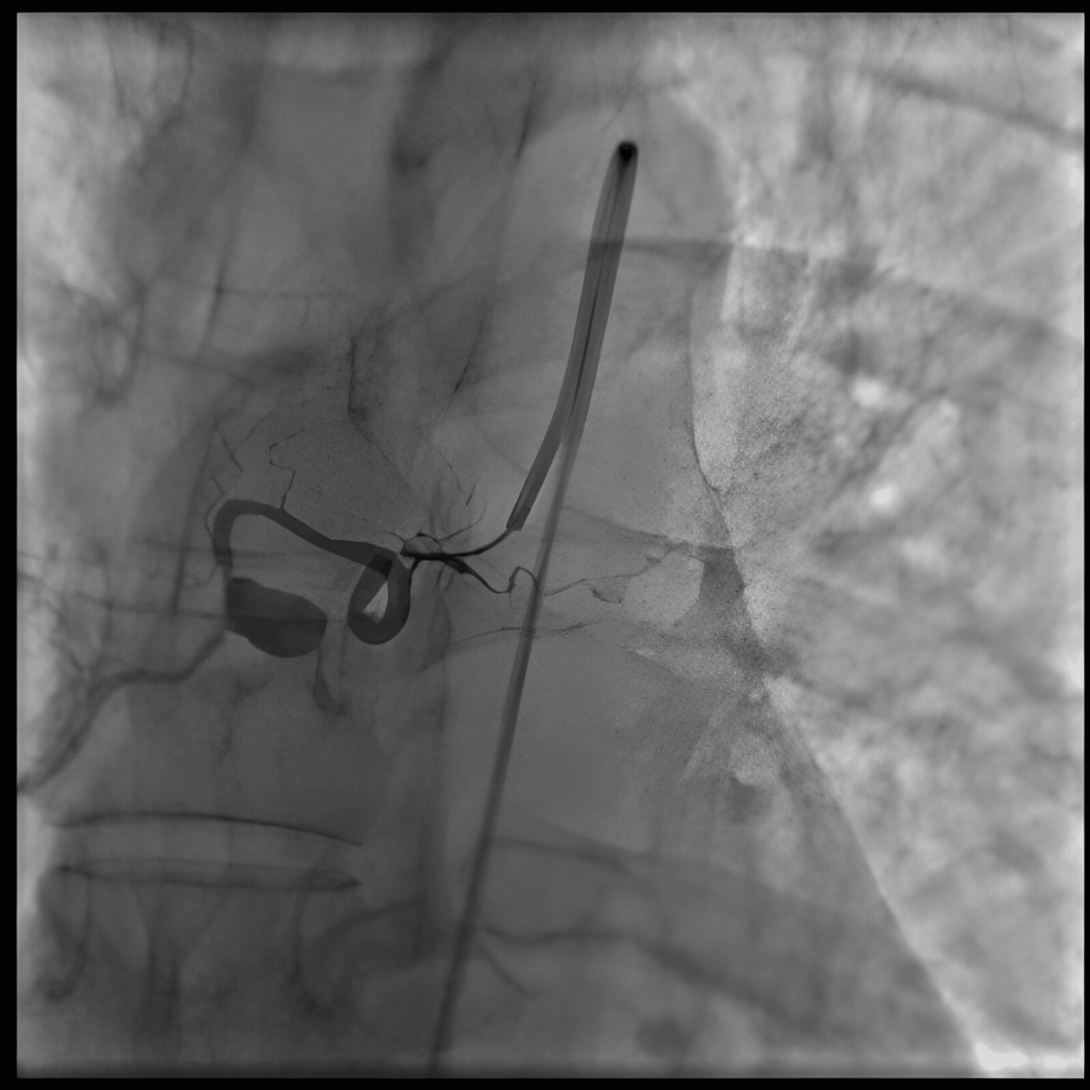
Selective bronchial artery angiography demonstrating a saccular pseudoaneurysm with associated contrast extravasation before embolization.

**Figure 7:**
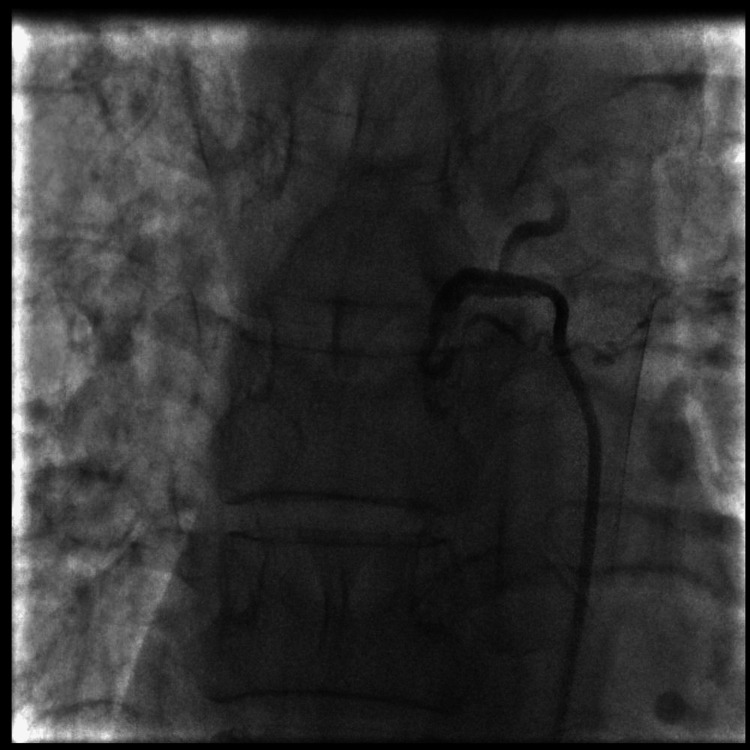
Post-embolization angiogram showing complete exclusion of the pseudoaneurysm and absence of contrast extravasation, confirming successful occlusion of the right bronchial artery.

Therapeutically, the patient first underwent emergency radiological embolization, which controlled the persistent gastrointestinal bleeding (Figures 6-7). Once the diagnosis was confirmed, a four-drug combination therapy for tuberculosis was initiated, combining isoniazid, rifampicin, pyrazinamide, and ethambutol during the intensive phase for four months, followed by a continuation phase with isoniazid and rifampicin for an additional four months. The outcome was favorable, marked by complete cessation of bleeding, gradual improvement in clinical and biological status, and good tolerance of the treatment. The patient is currently being monitored regularly in consultation, with satisfactory progress under treatment.

## Discussion

UGIB remains a frequent and potentially life-threatening emergency in gastroenterology. It is most commonly caused by peptic ulcer disease, variceal rupture secondary to portal hypertension, or severe erosive mucosal disease. However, atypical or rare etiologies, including vascular malformations, mediastinal aneurysms, and infectious processes, can also lead to severe bleeding episodes that are difficult to diagnose and manage [3].

The initial diagnostic approach to UGIB relies on upper endoscopy, which identifies the bleeding source in more than 90% of cases [4]. Yet, in certain situations, such as submucosal, extrinsic, or vascular lesions, endoscopic findings can be non-specific or even misleading. Negative or non-diagnostic biopsies, as in our case, may delay appropriate management. When conventional endoscopy fails to clarify the etiology, cross-sectional imaging (contrast-enhanced CT) and selective angiography become essential to evaluate mediastinal or vascular causes of bleeding [5,6].

Among rare causes, tuberculosis involving the mediastinum and esophagus represents an exceptional entity. Tuberculosis, caused by *Mycobacterium tuberculosis*, primarily affects the lungs, but extrapulmonary forms account for up to 20% of cases, particularly in endemic regions [7]. Mediastinal involvement typically arises from the reactivation of lymph node tuberculosis, with caseating necrosis, which leads to nodal enlargement and, occasionally, to rupture into adjacent structures, such as the esophagus or bronchial tree [7].

Esophageal tuberculosis is an exceptionally rare condition, first described in 1837, accounting for only about 0.1-0.2% of all tuberculosis cases [8]. It typically affects young adults, more often men, and remains uncommon even in HIV-positive patients [9]. It usually results from the direct extension of necrotic mediastinal lymph nodes. The middle third of the esophagus, anatomically adjacent to the subcarinal and paratracheal lymph nodes, is most frequently affected. Its rarity is attributed to the low permeability of the squamous esophageal mucosa, rapid peristaltic transit, efficient lymphatic drainage, and limited arterial supply [10].

The disease can manifest as dysphagia, odynophagia, retrosternal pain, or - more rarely - massive hematemesis. Bleeding may occur due to erosion of the esophageal wall into adjacent vascular structures, such as bronchial or intercostal arteries, or through ulceration of the mucosa overlying a necrotic lymph node [11].

Upper endoscopy is the key diagnostic tool, allowing visualization of the lesion and, crucially, biopsy sampling, since endoscopic appearances are non-specific and may mimic cancer, opportunistic infections, or Crohn’s disease [12].

Histopathological confirmation is challenging. Superficial endoscopic biopsies often fail to demonstrate granulomatous inflammation because the diagnostic material lies in the deeper layers of the wall or within adjacent lymph nodes. This explains why techniques like endobronchial ultrasound-guided transbronchial needle aspiration (EBUS-TBNA) or endoscopic ultrasound-guided fine needle aspiration (EUS-FNA) have gained importance in diagnosing mediastinal tuberculosis. These methods allow sampling of deep mediastinal or peri-esophageal lymph nodes with high sensitivity and safety [13,14].

In our case, the diagnosis was established only after EBUS-TBNA revealed necrotizing granulomatous inflammation consistent with tuberculosis, following non-diagnostic endoscopic biopsies. This diagnostic pathway aligns with previous studies reporting that even in symptomatic patients, classical endoscopic or radiological approaches may lack specificity or sufficient histological yield due to necrosis, hemorrhagic debris, or fibrosis.

Management of such complex cases requires both hemostatic control and etiologic treatment. When endoscopic hemostasis is not feasible, transarterial embolization offers a minimally invasive and effective alternative to control bleeding from bronchial or mediastinal arterial sources. Once the bleeding source is secured, initiation of anti-tuberculous therapy remains the cornerstone of management. Standard quadruple therapy, consisting of isoniazid, rifampicin, pyrazinamide, and ethambutol, typically leads to healing of granulomatous lesions, resolution of fistulae, and clinical improvement [15].

Prognosis depends largely on early detection and appropriate management, with mortality reaching up to 20% in immunocompetent individuals and 35% in HIV-positive patients [16,17].

## Conclusions

This case emphasizes the importance of maintaining a high index of suspicion for tuberculosis in the differential diagnosis of unexplained or refractory upper GI bleeding, especially in endemic areas. The coexistence of mediastinal lymphadenopathy, necrotic nodes, or inflammatory infiltration near the esophagus on imaging should prompt consideration of tuberculous involvement. Early recognition and a multidisciplinary approach involving gastroenterologists, radiologists, and infectious disease specialists are essential to prevent morbidity and recurrence.
